# Infección por SARS-CoV-2 en Receptores de Trasplante Cardíaco

**DOI:** 10.47487/apcyccv.v1i2.56

**Published:** 2020-06-29

**Authors:** Jhoel Yarahuaman-Mora, Juan Muñoz-Moreno, Walter Alarco-León

**Affiliations:** 1 Médico residente de Cardiología. Instituto Nacional Cardiovascular - INCOR EsSalud. Lima, Perú. Médico residente de Cardiología Instituto Nacional Cardiovascular - INCOR EsSalud Lima Perú; 2 Unidad de Falla Cardíaca, Trasplante Cardíaco e Hipertensión Pulmonar. Instituto Nacional Cardiovascular - INCOR EsSalud. Lima, Perú. Unidad de Falla Cardíaca, Trasplante Cardíaco e Hipertensión Pulmonar Instituto Nacional Cardiovascular - INCOR EsSalud Lima Perú

**Keywords:** trasplante cardíaco, SARS-CoV-2, COVID-19, cardiac transplant, SARS-CoV-2, COVID-19

## Abstract

Desde los primeros reportes de la infección por SARS-CoV-2 en el mes de diciembre de 2019 en Wuhan, China, hemos sido testigos de su propagación mundial y de las graves complicaciones que ha ocasionado en miles de pacientes. En la población de riesgo se encuentran los receptores de trasplantes de órganos sólidos quienes son especialmente vulnerables por su condición de inmunosuprimidos.

Aún no existe suficiente información sobre la presentación, curso clínico, manejo y pronóstico de la enfermedad por coronavirus (COVID-19) en estos pacientes.

Presentamos dos casos de pacientes trasplantados cardíacos con infección por SARS-CoV-2 atendidos en un instituto de Cardiología de la ciudad de Lima, Perú.

## Abstract (en)

Since the first reports of SARS-CoV-2 infection in December 2019 in Wuhan, China, we have witnessed its worldwide spread, and the serious complications it caused in thousands of patients. Recipients of solid organ transplants are among the risk population due to immunosuppression.

There is lack of information about the presentation, clinical course, management, and prognosis of coronavirus disease (COVID-19) in these patients.

We present 2 cases of heart transplant recipients who contracted SARS-CoV-2 infection and received treatment in a Cardiology Institute in Lima, Peru.

## Introducción

Desde los primeros reportes de la infección por el virus SARS-CoV-2 en el mes de diciembre de 2019 en Wuhan, China, hemos sido testigos de los graves efectos de esta pandemia. Su alta infectividad, morbilidad y mortalidad han ocasionado un colapso en los sistemas de salud de muchos países en el lapso de pocas semanas. 

El virus SARS-CoV-2 ingresa principalmente por el epitelio de las vías respiratorias utilizando la enzima convertidora de angiotensina 2 (ECA2) como receptor, y puede ocasionar desde una neumonía aguda leve, hasta casos graves como el síndrome de distrés respiratorio agudo severo (ARDS por sus siglas en inglés). ^(^^1^

Los receptores de trasplante cardíaco (TC) pueden tener mayor riesgo de desarrollar enfermedad grave por el virus SARS-CoV-2, ya que suelen tener múltiples comorbilidades y además se encuentran inmunosuprimidos. Además, se menciona que en ellos se prolonga la eliminación del virus.^1^^,^^2^^,^^3^^,^^4^^)^ Hasta la fecha, han sido muy limitados los reportes sobre la presentación, curso clínico y el pronóstico de la enfermedad por el nuevo coronavirus (COVID-19) en estos pacientes. ^(^^1^

Presentamos los casos de dos pacientes con antecedente de trasplante cardíaco que cursaron con infección por SARS-CoV2, hospitalizados en el Instituto Nacional Cardiovascular - INCOR EsSalud en Lima, Perú. En el reporte de ambos casos, haremos una descripción clínica desde el ingreso y su evolución a 30 días.

## Caso 1

Paciente de sexo masculino de 51 años, con antecedente de trasplante cardíaco ortotópico en enero del 2019 por cardiomiopatía dilatada no isquémica, y sin historia de rechazo del injerto desde el implante, que recibía terapia inmunosupresora de base con tacrolimus, micofenolato de mofetilo (MMF) y prednisona. 

Acudió al hospital con un tiempo de enfermedad de 10 días, caracterizado por tos seca, temperatura de 38°C, dia-rrea, disnea y taquipnea. Se realizó a su ingreso la prueba serológica (prueba rápida) para COVID-19, siendo esta positiva (IgM e IgG) para la infección.

**Figura 1:**
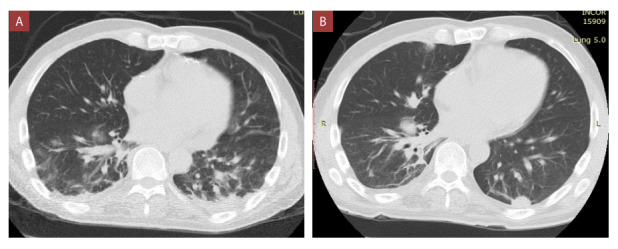
Tomografías torácicas sin contraste correspondientes al caso 1. **A)** Tomografía al ingreso muestra opacidades parcheadas en vidrio deslustroso, con tendencia a confluir en regiones periféricas de ambas bases pulmonares. **B)** Tomografía en el día 22 muestra resolución de las opacidades en vidro deslustroso y aparición de infiltrado intersticial tenue.

**Figura 2:**
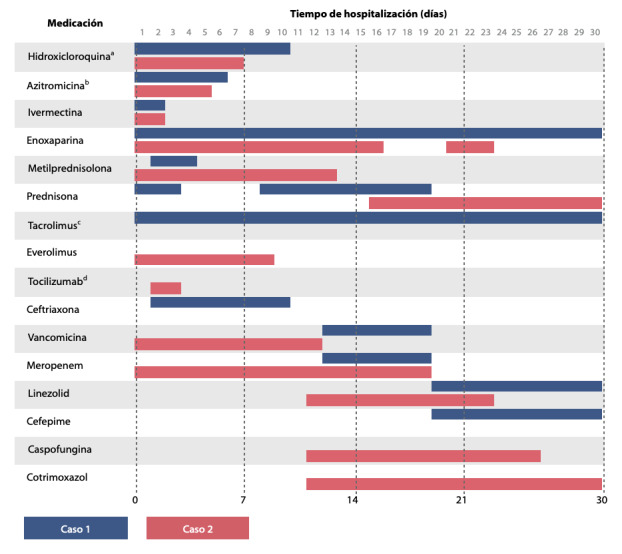
Medicación utilizada.

^a^ La primera dosis de hidroxicloroquina fue de 400 mg dos veces al día, posteriormente 200 mg dos veces al día. ^b^La dosis de azitromicina 500 mg el primer día, posteriormente 250 mg al día. ^c^La dosis de tacrolimus se ajustó de acuerdo al dosaje (día 2: 17.9 ng/mL; día 13: 3.54 ng/mL; día 26: 9.17 ng/mL). ^d^La dosis de tocilizumab fue de 600 mg el primer día y 200 mg el segundo día.

Ingresó al área COVID del hospital con frecuencia respiratoria (FR) 35 rpm y saturación de oxígeno (SO_2_) de 92%, por lo que se brindó apoyo oxigenatorio con cánula binasal (CBN) a 3 L/min. La analítica de ingreso se visualiza en la Tabla 1 y la tomografía de tórax en la Figura 1. Con esos datos se catalogó como un cuadro moderado (marcadores pronósticos elevados) y se inició esquema de tratamiento de acuerdo con la Figura 2. Se suspendió desde el ingreso el MMF y se redujo dosis de tacrolimus a un 50% con el dosaje al 2do día de ingreso (17.9 ng/mL). La evolución de los valores de tacrolimus durante la hospitalización se mencionan en la descripción de la Figura 2. 

El paciente permaneció afebril desde el segundo día de hospitalización y se le indicó pronación consciente con disminución progresiva de la necesidad de oxígeno por CBN. Al día 16 de hospitalización y con dos pruebas serológicas negativas separadas una de otra en 48 horas, se decide transferencia a un área no COVID. Posteriormente, por un incremento de temperatura, alteraciones de laboratorio y estudios de imágenes, se evidenció un proceso neumónico asociado, por lo que recibió meropenem y vancomicina por 7 días. Luego se cambió a terapia con linezolid y cefepime ante la alta sospecha de neumonía necrotizante. La evolución clínica fue favorable al mes de hospitalización, y se dio el alta médica sin complicaciones.

Cabe mencionar que el paciente previo al ingreso presentaba una fracción de eyección del ventrículo izquierdo (FEVI) preservada, que no se alteró durante la hospitalización. Tampoco hubo elevación de enzimas cardíacas. No se detectó carga viral para citomegalovirus y el estudio del bacilo de Koch (BK) en esputo fue negativo.

## Caso 2

Paciente de sexo masculino de 63 años, con ante-cedente de trasplante cardíaco ortotópico en octubre del 2017 por cardiomiopatía dilatada isquémica e historia de rechazo celular 2R el año 2018. Asimismo, padecía diabetes mellitus tipo 2 e insuficiencia renal crónica estadio 2. La terapia inmunosupresora consistía en everolimus, MMF y prednisona a dosis baja (5 mg al día). 

Acudió al hospital con un tiempo de enfermedad de 7 días, presentando rinorrea, tos seca, fiebre y disnea. Se realizó prueba serológica a su ingreso que resultó positiva para COVID-19 (IgM). Ingresó al área COVID del hospital con FR 24 rpm y SatO_2_ 82%, y se le otorgó apoyo oxigenatorio por CBN a 5 L/min.

**Figura 3:**
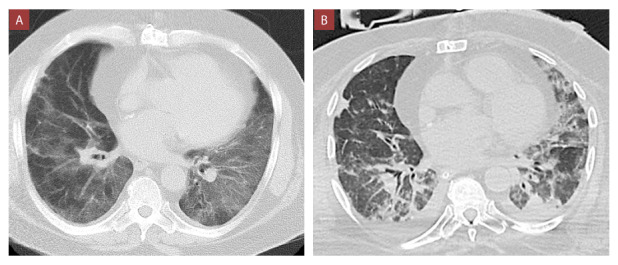
Tomografías torácicas correspondientes al caso 2.

Al examen físico se evidenció crepitantes en tercio inferior de ambos hemitórax. La analítica sanguínea se visua-liza en la Tabla 1, y la tomografía de tórax, en la Figura 3. Por el cuadro infeccioso asociado a congestión pulmonar y la pre-sencia de marcadores de mal pronóstico, se catalogó como neumonía aguda severa asociada a falla cardíaca aguda-mente descompensada. Se procedió a intubación orotraqueal y ventilación mecánica, e inició tratamiento de acuerdo con la Figura 2. Presentó linfopenia severa y leucocitosis marcada en ascenso progresivo los primeros días, pero con tendencia al descenso durante la evolución. Recibió linezolid y caspofungina debido a infección bacteriana y micótica. Además, desde el ingreso se suspendió MMF, everolimus y prednisona. Desde el punto de vista ventilatorio, requirió ventilación prona al día 3, 10 y 16, por 72 hrs en cada periodo. 

**Tabla 1:** Exámenes auxiliares durante la hospitalización

	Caso 1				Caso 2			
	Ingreso	Día 7	Día 30		Ingreso	Día 7	Día 30	
Hemograma								
Hemoglobina (gr/dL)	11.4	11.8	9.1		13.6	12.2	8.7	
Leucocitos (x10^3^/uL)	6.13	4.41	4.6		16.07	25.93	9.74	
Linfocitos (x10^3^/uL)	1.02	1.36	1.09		0.44	0.44	0.62	
Plaquetas (x10^3^/uL)	182	274	389		170	319	118	
Bioquímico								
TGO (U/L)	98	63	22		29	35	34	
TGP (U/L)	63	142	30		27	43	83	
Urea (mg/dL)	58	55	43		55	170	58	
Creatinina (mg/dL)	0.73	0.76	0.81		1.75	1.79	0.98	
Lactato deshidrogenasa (U/L)	589	323	254		578	471	1192	
Dimero D (mcg/mL)	0.47	0.6	1.4		0.28	1.02	1.14	
Fibrinógeno (mg/dL)	427	414	−		1183	664	507	
Ferritina (ng/mL)	875.5	730.2	−		−	896.4	391.5	
Proteina C reactiva (mg/L)	36.9	4.2	7		365.8	20	21	
Procalcitonina (ng/mL)	0.13	−	0.06		0.38	0.08	0.38	
Gasometría								
pH	7.45	7.41	−		7.42	7.4	7.42	
pO_2_ (mmHg)	73	77.7	−		70.2	91	83.9	
pCO_2_ (mmHg)	27.1	31.4	−		33.2	43	42.6	
Sodio (mEq/L)	127	134	−		132	151	138	
Potasio (mEq/L)	4.2	4.2	−		4.4	4.1	3.8	
Lactato (mmol/L)	0.5	2.1	−		1.3	1	1	
PaFiO_2_ (mmHg)	348	323	−		195	260	209	

TGO: transaminasa glutámico-oxalacética; TGP: transaminasa glutámico-pirúvica

A) Tomografía al ingreso muestra opacidades confluentes en vidrio deslustroso visualizados en regiones periféricas y centrales de ambas bases pulmonares. B) Tomografía en el día 24 muestra bandas parenquimales desorganizadas y escasas áreas en vidrio deslustroso en ambas bases pulmonares a predominio del lado izquierdo y leve efusión pleural bilateral.

En la evolución desarrolló injuria renal aguda AKIN 3 (día 12), sin indicación de terapia de reemplazo renal y con remisión posterior. Presentó también hematoma periumbi-lical extenso y caída de hemoglobina que requirió suspensión de la anticoagulación (entre el día 17 y 20). Se complicó con un accidente cerebrovascular (ACV) isquémico parieto-occipital derecho evidenciado en tomografía cerebral al día 24 (solicitada por anisocoria). Finalmente, se realizó traqueos-tomía percutánea tras 1 mes de intubación orotraqueal. 

El paciente presentaba una FEVI preservada previo al ingreso, y durante hospitalización se apreció una FEVI estimada de 45%, con ligero incremento de enzimas cardíacas. No se pudo descartar rechazo agudo del injerto ni realizar biopsia endomiocárdica por inestabilidad hemodinámica e injuria renal aguda. Durante estos días se mantuvo metilprednisolona en desescalamiento progresivo gradual (40 mg endovenoso una vez al día). 

El pronóstico al momento de la redacción de este artículo es incierto debido a la evolución tórpida del paciente. Actualmente cursa con un proceso infeccioso pulmonar sobreagregado, que en la tomografía de tórax de control se manifiesta con imágenes consolidativas desorganizadas, leve efusión pleural bilateral, asociado a disminución de las áreas en vidrio deslustroso.

## Discusión

El SARS-CoV2, un nuevo tipo de coronavirus, surgió en Wuhan, China, en diciembre de 2019. Desde entonces, se han generado muchas dudas en cuanto a la fisiopatología, diagnóstico y manejo. Dentro de lo conocido al momento, afecta con más frecuencia y con mayor grado de complicaciones a aquellas personas con comorbilidades como diabetes mellitus, hipertensión arterial, obesidad e inmunosupresión. En este último grupo, se encuentran los pacientes trasplantados.^5^ Sin embargo, a pesar de múltiples publicaciones, no se ha reportado si la condición de trasplantado representa un mayor riesgo de infección por el virus o una mayor susceptibilidad de desarrollar cuadros severos; no obstante, podría incrementarse ese riesgo si están asociadas comorbilidades. Entre los pocos artículos publicados, Ren et al.^1^ reportan la experiencia de 87 pacientes trasplantados cardíacos en Hubei, China. 57 de ellos tenían historia de viajes recientes a Wuhan o contacto con residentes de Wuhan y sin embargo, ninguno desarrolló la enfermedad. Una explicación a sus hallazgos es que algunos de dichos pacientes (10 de 47 pacientes muestreados) ya tenían cierta linfopenia producto del uso de inmunosupresores, evitando el desarrollo de linfocitos y citoquinas pro-inflamatorias. Además, el uso de corticoides refuerza este efecto, por lo que los pacientes trasplantados podrían no exhibir cuadros típicos.^1^

Li et al.^6^ reportan 2 casos de pacientes trasplantados cardíacos con COVID-19, con clínica de fiebre, fatiga, disminución de apetito y diarrea, sin ninguna complicación ni necesidad de ingreso a una unidad de cuidados intensivos (UCI). Ambos pacientes recibieron umifenovir 200 mg tres veces por día y ganciclovir 250 mg/día por 7 días. El primer paciente además recibió gammaglobulina humana 10 gr/día y metilprednisolona 80 mg/día por 5 días. Las imágenes tomográficas fueron muy similares a las de los pacientes reportados en este artículo, y al momento del alta (aproximadamente a las dos semanas) dichas imágenes no mostraron resolución completa a pesar del alivio sintomático.^6^

Latif et al.,^8^ en un grupo de 28 pacientes trasplantados cardíacos con el diagnóstico de COVID-19, reportaron que 22 requirieron ingreso hospitalario, teniendo como principales co-morbilidades hipertensión arterial, diabetes mellitus, obesidad y enfermedad renal crónica. 7 (25%) necesitaron ingreso a UCI, y 7 (25%) fallecieron, posiblemente debido al alto porcentaje de comorbilidades asociadas, que favorecieron el desarrollo de cuadros graves. 

En relación con los esquemas de inmunosupresión crónica de estos pacientes, dependiendo de la severidad del caso, se recomienda suspender el agente anti-proliferativo (MMF), reducir la dosis del anti-calcineurínico (ciclosporina, tacrolimus) en casos leves y moderados o suspenderlo en los severos. En lo posible se debería mantener por lo menos los esteroides de manera parenteral empleando generalmente metilprednisolona.^3^ Es importante el dosaje del anticalci-neurínico para el reajuste de dosis, debido a que se pueden presentar interacciones farmacológicas con fármacos como la hidroxicloroquina, azitromicina o antibióticos. En el caso de nuestro primer paciente, luego de la reducción inicial de la dosis de tacrolimus, se evidenció una disminución significativa de sus valores en sangre, explicada probable-mente por esta interacción.

Hasta el momento no existe un protocolo consen-suado o terapia eficaz para este grupo de pacientes, y las recomendaciones de manejo se basan en la experiencia encontrada en la población no trasplantada. ^(^^9^ La hidroxi-cloroquina, por su acción inmunomoduladora y su probable efecto sobre la tormenta de citoquinas, ^(^^5^^)^ y la azitromicina, fueron las primeras terapias utilizadas. Sin embargo, existe un alto riesgo de prolongar el intervalo QT e incrementar los niveles de anticalcineurínicos con estos medicamentos. Por lo tanto, es importante monitorizar el electrocardiograma día a día y realizar el dosaje del anticalcineurínico como fue men-cionado anteriormente. Al momento de realizar este reporte, no existen estudios con algún grado de evidencia que respalden el uso de los medicamentos descritos; sin embargo, en el Perú la administración de estos se dio bajo las recomendaciones de la resolución ministerial Nº 193-2020 del Ministerio de Salud (MINSA). ^(^^12^

El tocilizumab, un inhibidor de interleuquina 6 (IL6), es utilizado en algunos protocolos en los casos con neumonía aguda severa, cuando existe una respuesta inflamatoria sistémica marcada, buscando inhibir la tormenta de cito-quinas. Su uso en pacientes trasplantados tendría la misma indicación y cuidados que en la población general. ^(^^13^ Sin embargo, deberíamos tener mayor cuidado con las sobre-infecciones, como pudiera ser el caso de nuestro segundo paciente.

La ivermectina ha demostrado en algunos estudios su eficacia in vitro, pero se cuestiona la misma in vivo, ya que alcanza una concentración baja en tejido pulmonar (órgano diana de compromiso en el COVID-19). Dosis de incluso 10 veces la dosis máxima aceptada llegan a solo una cuarta parte de la concentración inhibitoria al 50% (CI50) que sería la mínima requerida para lograr algún efecto contra patógenos. Además, este medicamento tiene efectos adversos neurológicos por su facilidad para atravesar la barrera hematoencefálica, produciendo somnolencia, náuseas e incluso midriasis en dosis por encima de las recomendadas. ^14^

La anticoagulación con heparina presenta propie-dades adyuvantes al bloquear la formación de trombina (inflamación y formación de trombina son eventos directa-mente relacionados). ^(^^15^^,^^16^ En ambos pacientes se administró enoxaparina, una heparina de bajo peso molecular (HBPM), a dosis dosis plena de 1 mg/kg subcutáneo cada 12 hrs por presencia de marcadores de inflamación elevados y alto riesgo tromboembólico, sobre todo en el caso 2 (movilidad reducida, índice de masa corporal > 30). Se recomienda además prolongar el uso de la HBPM en dosis profilácticas durante 7-10 días tras el alta._^(17)^_

## Conclusiones

Los pacientes trasplantados constituyen una pobla-ción vulnerable y en riesgo para la infección por el virus SARS-CoV-2. Los marcadores laboratoriales de riesgo estuvieron elevados en ambos casos. Se brindó soporte ventilatorio según la necesidad de cada caso y maniobras de rescate como la pronación. La terapia inmunosupresora tuvo que ser regulada en ambos casos. Se utilizó un esquema de manejo en base a la resolución ministerial del MINSA sobre la prevención y atención de personas infectadas por COVID-19 en el Perú; teniendo en cuenta que la anticoagulación, las maniobras de pronación y un adecuado manejo de la ventilación mecánica en los casos severos son las estrategias más importantes de tratamiento.

Las características clínicas, marcadores de inflamación elevados y los estudios de imágenes de nuestros pacientes mantienen las características de la población general infectada por SARS-CoV-2. No podemos concluir que la condición de trasplantado predispone a cuadros severos, debido al reporte de sólo dos casos.

## Financiación:

Ninguna
